# Urinary TYROBP and HCK as genetic biomarkers for non-invasive diagnosis and therapeutic targeting in IgA nephropathy

**DOI:** 10.3389/fgene.2024.1516513

**Published:** 2024-12-24

**Authors:** Boji Xie, Shuting Pang, Yuli Xie, Qiuyan Tan, Shanshan Li, Mujia Jili, Yian Huang, Binran Zhao, Hao Yuan, Junhao Mi, Xuesong Chen, Liangping Ruan, Hong Chen, Xiaolai Li, Boning Hu, Jing Huang, Rirong Yang, Wei Li

**Affiliations:** ^1^ Department of Nephrology, The Second Affiliated Hospital of Guangxi Medical University, Nanning, Guangxi, China; ^2^ Guangxi Key Laboratory for Genomic and Personalized Medicine, Center for Genomic and Personalized Medicine, Guangxi Collaborative Innovation Center for Genomic and Personalized Medicine, University Engineering Research Center of Digital Medicine and Healthcare, Guangxi Medical University, Nanning, Guangxi, China; ^3^ Department of Immunology, School of Basic Medical Sciences, Guangxi Medical University, Nanning, Guangxi, China; ^4^ Medical Laboratory Department, Liuzhou Maternity and Child Healthcare Hospital, Liuzhou, China

**Keywords:** IgA nephropathy, urine bulk RNA sequencing, non-invasive biomarkers, TYROBP, HCK, molecular docking

## Abstract

**Background:**

IgA nephropathy (IgAN) is a leading cause of renal failure, but its pathogenesis remains unclear, complicating diagnosis and treatment. The invasive nature of renal biopsy highlights the need for non-invasive diagnostic biomarkers. Bulk RNA sequencing (RNA-seq) of urine offers a promising approach for identifying molecular changes relevant to IgAN.

**Methods:**

We performed bulk RNA-seq on 53 urine samples from 11 untreated IgAN patients and 11 healthy controls, integrating these data with public renal RNA-seq, microarray, and scRNA-seq datasets. Machine learning was used to identify key differentially expressed genes, with protein expression validated by immunohistochemistry (IHC) and drug-target interactions explored via molecular docking.

**Results:**

Urine RNA-seq analysis revealed differential expression profiles, from which *TYROBP* and *HCK* were identified as key biomarkers using machine learning. These biomarkers were validated in both a test cohort and an external validation cohort, demonstrating strong predictive accuracy. scRNA-seq confirmed their cell-specific expression patterns, correlating with renal function metrics such as GFR and serum creatinine. IHC further validated protein expression, and molecular docking suggested potential therapeutic interactions with IgAN treatments.

**Conclusion:**

*TYROBP* and *HCK* are promising non-invasive urinary biomarkers for IgAN. Their predictive accuracy, validated through machine learning, along with IHC confirmation and molecular docking insights, supports their potential for both diagnostic and therapeutic applications in IgAN.

## Introduction

IgAN is a leading cause of primary glomerulonephritis and renal failure worldwide, with pathological features such as mesangial proliferation (Cai and Chen, 2009; Roberts, 2014). Classified as mesangial proliferative glomerulonephritis (MsPGN) (Wehbi et al., 2019), IgAN is particularly prevalent in the Asia-Pacific region, where it accounts for 30%–40% of primary glomerular diseases in China, imposing significant economic and psychological burdens (Cai and Chen, 2009; Magistroni et al., 2015). Renal biopsy remains the gold standard for diagnosing IgAN; however, it is invasive, carries risks, and is subject to variability based on pathologist interpretation. Recent advances in non-invasive diagnostic techniques, particularly liquid biopsy methods using urine biomarkers, offer great promise (Beckmann et al., 2020; Soliman et al., 2022; Chen et al., 2023a). These biomarkers include microRNAs (Levstek et al., 2023), small metabolites (Chen et al., 2023b), cytokines (Cambier et al., 2022), and collagens (Sparding et al., 2023a; Sparding et al., 2023b), which could complement or replace invasive procedures in clinical settings.

Urine, as a direct product of kidney function, holds distinct advantages for non-invasive diagnosis. It reflects both physiological and pathological states of the kidneys, making it invaluable for disease monitoring, diagnosis, and therapeutic evaluation (Wang et al., 2021). Compared to blood, urine offers easier collection and heightened sensitivity in detecting kidney-related molecular changes, providing a clearer platform for early detection and disease progression monitoring (Li et al., 2014; Gao, 2013). Therefore, urinary biomarkers represent a promising tool for enhancing the early identification and management of IgAN.

Notably, previous urinary studies have predominantly focused on first morning urine samples (Tuttle et al., 2024; Masone, 2024). However, recent investigations have increasingly recognized the diagnostic and metabolic relevance of second morning urine samples (Liu et al., 2020; Segawa et al., 2021). In this study, we hypothesized that cells present in second morning urine samples may more accurately reflect renal function, as epithelial cells from the urinary tract are likely to have been “flushed out” during the initial void. To test this, we performed bulk RNA-seq on urinary cells from IgAN patients, including first morning, second morning, and random urine samples. By integrating our internal urinary bulk RNA-seq data with publicly available IgAN datasets, and utilizing machine learning analyses alongside immunohistochemical validation, we identified *TYROBP* and *HCK* as potential non-invasive urinary biomarkers for the diagnosis of IgAN. These findings align with previous studies, which have also identified TYROBP and HCK as promising biomarkers for IgAN, using bioinformatics approaches (Bai et al., 2022; Li et al., 2023).

## Materials and methods

### Urine sample collection and cell isolation

This study included 11 treatment-naive patients diagnosed with IgAN via renal biopsy and 11 age- and sex-matched healthy controls. Exclusion criteria encompassed malignancies, severe liver dysfunction, and other rheumatic diseases. Inclusion criteria required: 1) no prior drug therapy or renal tissue puncture before urine collection; 2) IgAN diagnosis based solely on post-collection renal biopsy. All biopsies were evaluated by pathologists blinded to patient outcomes; 3) signed informed consent.

Participants provided three urine samples: first morning, second morning, and a random sample. Urine samples were promptly transferred to 50 mL centrifuge tubes and centrifuged at 490 × g for 10 min. The supernatant was discarded, and the tubes were rinsed with Dulbecco’s phosphate-buffered saline (DPBS) to recover preliminary urine cells. These cells were then centrifuged at 2000 rpm for 5 min, with the supernatant removed and the process repeated twice. The resultant urine cell pellets were stored at −80°C for subsequent analysis. All procedures were carried out on ice to preserve sample integrity.

### Urine cell reverse transcription, library construction, and bulk RNA sequencing

Urine cells were lysed by adding 30 μL of lysis buffer, followed by centrifugation at 10,000 r/min. The lysate was incubated at room temperature for 6 min. Two microliters of the supernatant were extracted and promptly added to the OneStep reaction system (AccuraCode^®^ HTP OneStep RNAseq Kit, Singleron, Nanjing, China) while maintained on ice. The mixture was homogenized by pipetting and subjected to reverse transcription in a PCR machine. After reverse transcription, the products were purified and quality-checked. Subsequent steps included fragmentation, adapter ligation, purification of ligated products, PCR enrichment, and library sorting to complete library construction. Libraries that passed quality control were then subjected to bulk RNA sequencing.

### Online dataset acquisition

This study incorporated 10 independent datasets. Two RNA-seq datasets for IgA nephropathy (n = 41, IgAN = 31, control = 10) were obtained from the National Center for Biotechnology Information (NCBI) Gene Expression Omnibus (GEO) database (https://www.ncbi.nlm.nih.gov/geo/) with accession numbers GSE141295 and GSE210098. Additionally, four microarray datasets for IgAN (n = 170, IgAN = 100, control = 70) were downloaded from GEO with accession numbers GSE37463, GSE93798, GSE99340, and GSE104948. Finally, four scRNA-seq datasets were obtained from GEO: GSE131685 (n = 3, control = 3), GSE171314 (n = 5, IgAN = 4, control = 1), GSE127136 (n = 29, IgAN = 18, control = 11), and GSE140989 (n = 24, control = 24). Specific details regarding each dataset can be found in Supplementary Table 1.

### Analysis of bulk RNA sequencing data

In the analysis of bulk RNA-seq data, the R package sva (version 3.52.0) was used to remove batch effects between samples. Differential expression analysis between normal and IgAN samples in urine and kidney tissue was conducted using the R package edgeR (version 3.42.4) with thresholds of |logFC| > 1 and *p*-value <0.05. The differentially expressed genes (DEGs) from the three urine sample types were then subjected to an intersection analysis, focusing on genes upregulated only in the second morning urine. These genes were further intersected with those upregulated in IgAN kidney tissue, resulting in a set of genes that are jointly upregulated in both the second morning urine and kidney tissue of IgAN patients. These genes are considered to accurately reflect the kidney’s status. Venn diagrams were generated using the R package VennDiagram (version 1.7.3), and ROC curves were plotted using the R package pROC (version 1.18.5).

### Predictive feature construction using ensemble machine learning

To build a robust predictive model with high accuracy, machine learning analysis was performed using the R packages glmnet, caret, and xgboost. Initially, Lasso and Ridge regression were conducted using glmnet to select features, and data preprocessing and model training were performed using the caret package. Cross-validation was employed to optimize model parameters and prevent overfitting. Subsequently, gradient boosting decision trees (GBDT) were trained using the xgboost package to enhance the model’s predictive performance and robustness. An ensemble model was then constructed by combining predictions from multiple models, further improving accuracy and reliability. These steps ensured the reliability and efficiency of the constructed model.

### Analysis of microarray data

In analyzing microarray data, the R package sva (version 3.52.0) was used to remove batch effects, ensuring data accuracy. The R package pROC (version 1.18.5) was then utilized to plot ROC curves, evaluating the classification performance and predictive efficacy of the models.

### Analysis using nephroseq v5 database

Data were downloaded from the Nephroseq v5 renal disease database (http://v5.nephroseq.org/) and subjected to correlation analysis. Scatter plots were generated to illustrate relationships between variables, while box plots were used to display data distribution and differences between groups. Pearson’s correlation coefficient was used to assess the relationship between clinical data and gene expression levels. Differences in gene expression levels between groups were evaluated using Wilcoxon tests. A *p-value* < 0.05 was considered statistically significant.

### Single cell RNA sequencing data analysis

ScRNA-seq data were analyzed using the Seurat package (version 5.1.0). Quality control was performed by filtering cells with mitochondrial gene content below 30%, and highly variable genes expressed in at least 3 cells within the 200–2,500 expression range were selected for further analysis. Batch effects were removed using the Harmony package. Cell clusters were constructed using the “FindClusters” and “FindNeighbors” functions and visualized using the t-SNE method. Cells were annotated based on marker genes of different cell types. Gene expression distributions in single-cell RNA-seq data were visualized using the Nebulosa package. Additionally, cell development trajectories and fate decisions in scRNA-seq data were analyzed using the monocle package.

### KEGG and GO enrichment analysis

Gene Ontology (GO) enrichment and Kyoto Encyclopedia of Genes and Genomes (KEGG) pathway analysis were performed using the R package clusterProfiler (version 4.10.0). GO analysis identified significantly enriched biological processes, molecular functions, and cellular components, while KEGG pathway analysis revealed significant enrichment of target genes in metabolic and signaling pathways. A *p-value* < 0.05 was considered statistically significant, ensuring the reliability of the enrichment results.

### Pseudotime analysis

Pseudotime analysis of scRNA sequencing data from Seurat objects was performed using Monocle 2 (version 2.30.1). RNA count matrices were extracted, and a CellDataSet object was created. After normalization and dispersion estimation, highly variable genes were selected. Dimensionality reduction was conducted using the DDRTree method, and pseudotime trajectories were constructed to reveal developmental pathways.

### Immunohistochemistry staining

IgAN and normal kidney tissues were fixed in 4% paraformaldehyde for 48 h and subsequently embedded in paraffin. The paraffin-embedded tissues were sectioned into 3-μm-thick slices, followed by deparaffinization and rehydration. Antigen retrieval was performed by heating the sections in EDTA solution, first at medium heat for 8 min until boiling, then at medium-low heat for an additional 7 min. To block endogenous peroxidase activity, the sections were treated with 3% hydrogen peroxide and incubated in the dark at room temperature for 25 min. Non-specific binding was minimized by coating the sections with 3% BSA, followed by a 30-min blocking step at room temperature. After removing the blocking solution, primary antibodies—anti-TYROBP (Zenbio, R383147, 1:100) and anti-HCK (Proteintech, 11600-1-AP, 1:50)—were applied and incubated overnight at 4°C. The following day, sections were incubated with HRP-conjugated secondary antibodies for 50 min at room temperature. Visualization was achieved using diaminobenzidine (DAB) staining. Integrated optical density (IOD) analysis of IHC staining was performed using ImageJ software to quantify the expression levels of TYROBP and HCK in IgAN and normal kidney tissues.

### Screening and docking of drugs

We initially utilized the Drug Signatures Database (DSigDB) to predict potential small molecules that interact with our selected genes. Following this, we employed AutoDock for molecular docking to explore these interactions. The molecular structures of the selected genes were obtained from the PDB database (https://rcsb.org/), while the biomolecular structures of the targets were downloaded from the PubChem database (https://pubchem.ncbi.nlm.nih.gov/).

Adhering to standard docking protocols, we performed automated docking simulations between the biomolecules and the small molecules. The strength of the interactions was assessed based on the lowest binding energy. Visualization of the docking results was conducted using CB-Dock2.

### Statistical analysis

All statistical analyses were performed using R (version 4.3.1). A *p*-value <0.05 was considered statistically significant. Detailed descriptions of additional statistical tools, methods, and thresholds are provided within the methods section.

## Results

### Differentially expressed genes in the urine of IgAN patients reveal specific upregulation in second morning urine samples

**FIGURE 1:**
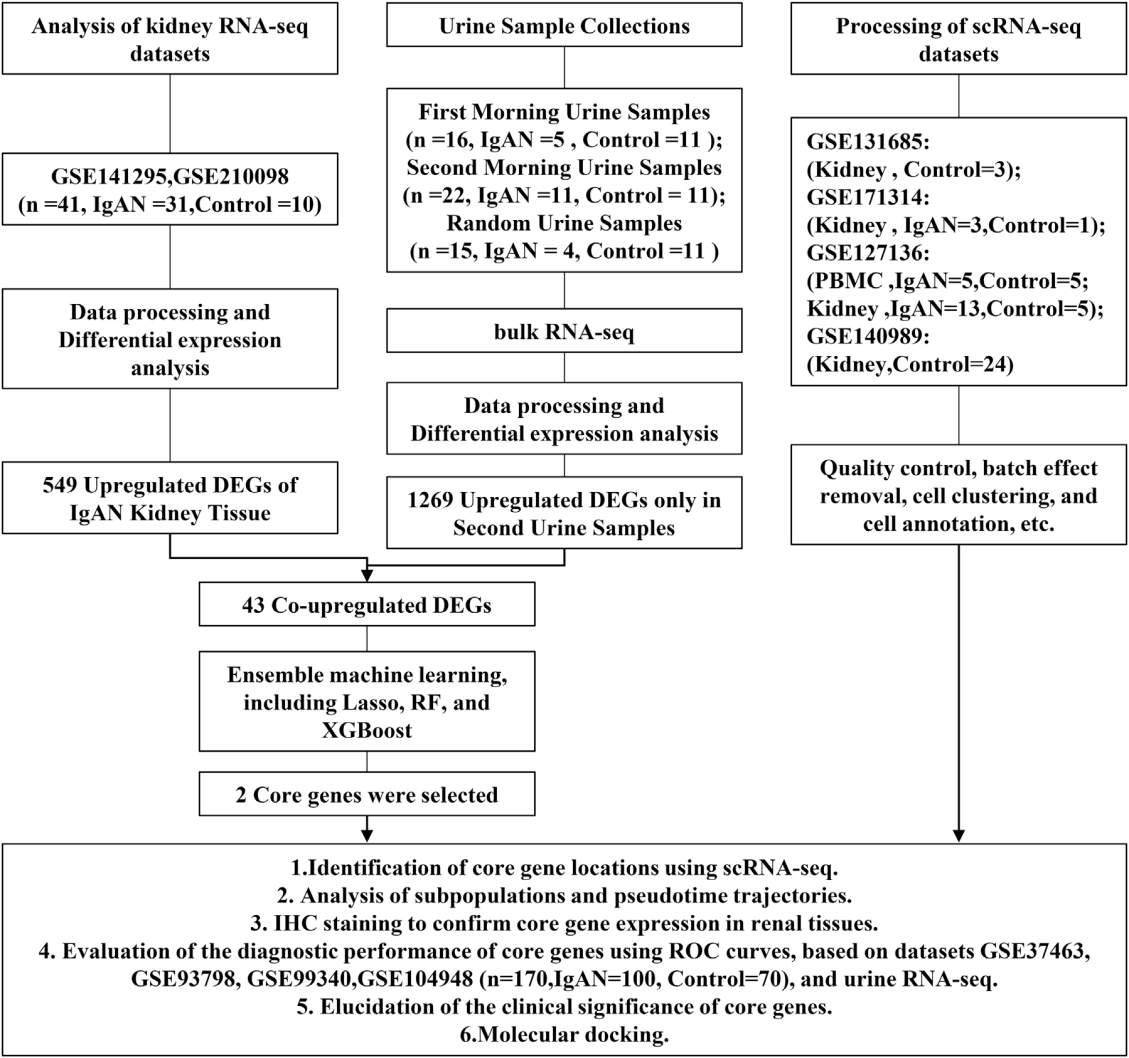
Workflow diagram.

**FIGURE 2:**
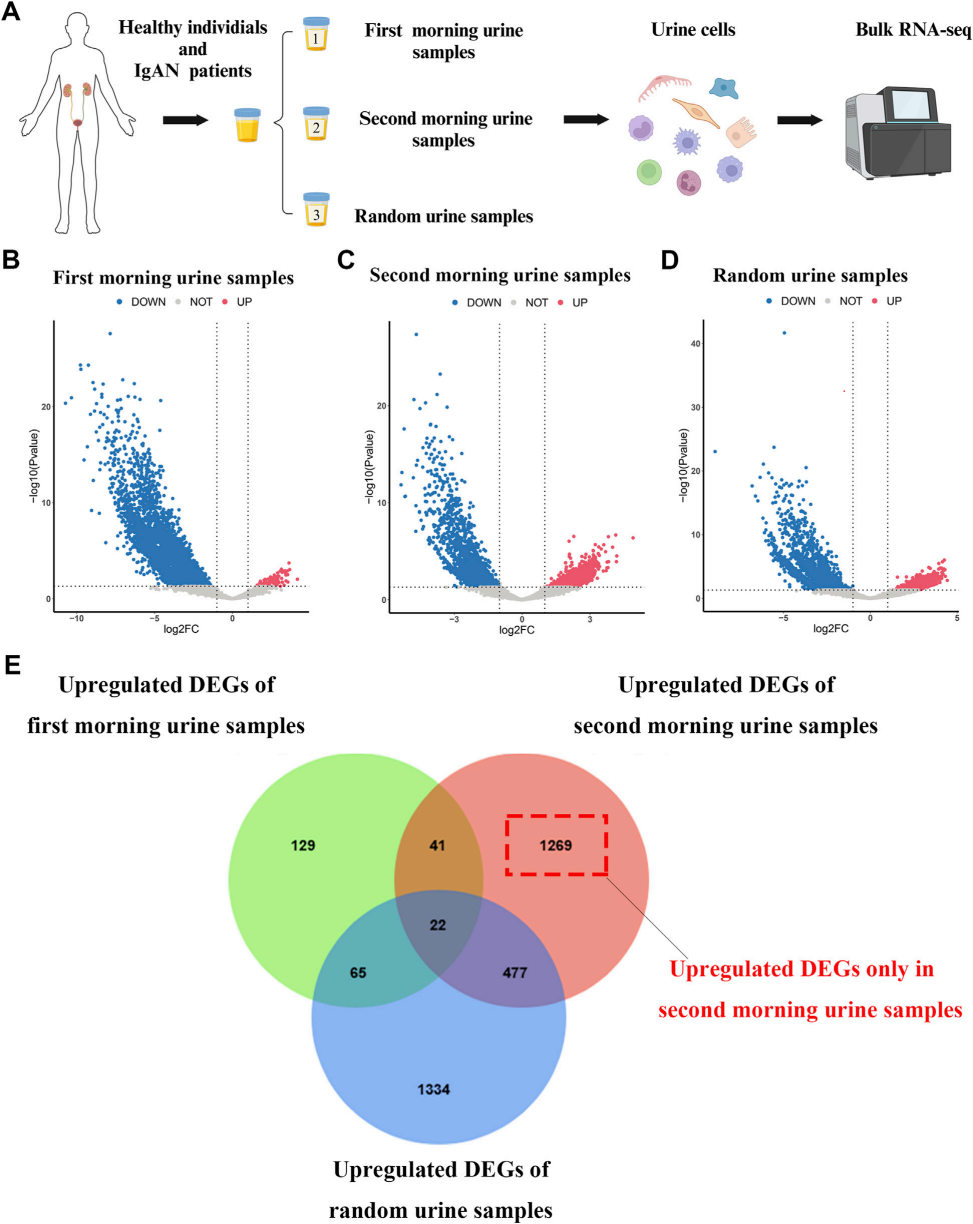
Workflow for urine cell sampling, bulk RNA sequencing, and differential analysis **(A)** Workflow diagram depicting the processing of urine samples and the bulk RNA sequencing procedure. **(B–D)** Volcano plots illustrating differential gene expression between IgAN patients and healthy controls in first morning urine, second morning urine, and random urine samples. **(E)** Venn diagram showing the overlap of upregulated differential genes between IgAN patients and healthy controls across first morning urine, second morning urine, and random urine samples.

The overall research approach is outlined in Figure 1. In this study, we collected morning urine, second morning urine, and random urine samples from 11 treatment-naïve IgAN patients and 11 healthy volunteers. Detailed baseline characteristics of the patients are provided in Supplementary Table 2. Following reverse transcription and library preparation, bulk RNA-seq was performed on urine-derived cells, and subsequent data analyses were conducted (Figure 2A). After processing, the final analysis included 5 First morning urine samples, 11 second morning urine samples, and 4 random urine samples from the IgAN group, as well as 11 samples of each type from the healthy control group.

To investigate the differential gene expression in the urine of IgAN patients, we performed differential expression analysis on morning urine, second morning urine, and random urine between the IgAN and healthy control groups (Figures 2B–D). The results revealed 257 upregulated genes in morning urine, 1809 in second morning urine, and 1898 in random urine (Supplementary File 1). Intersection analysis identified 1,269 genes that were specifically upregulated only in second morning urine (Figure 2E). These specific genes (Supplementary File 2) are likely more reflective of the pathological changes in IgAN and may hold potential as biomarkers.

### Renal origin and functional annotation of specific upregulated genes in second morning urine samples point to their relevance in IgAN pathology

**FIGURE 3:**
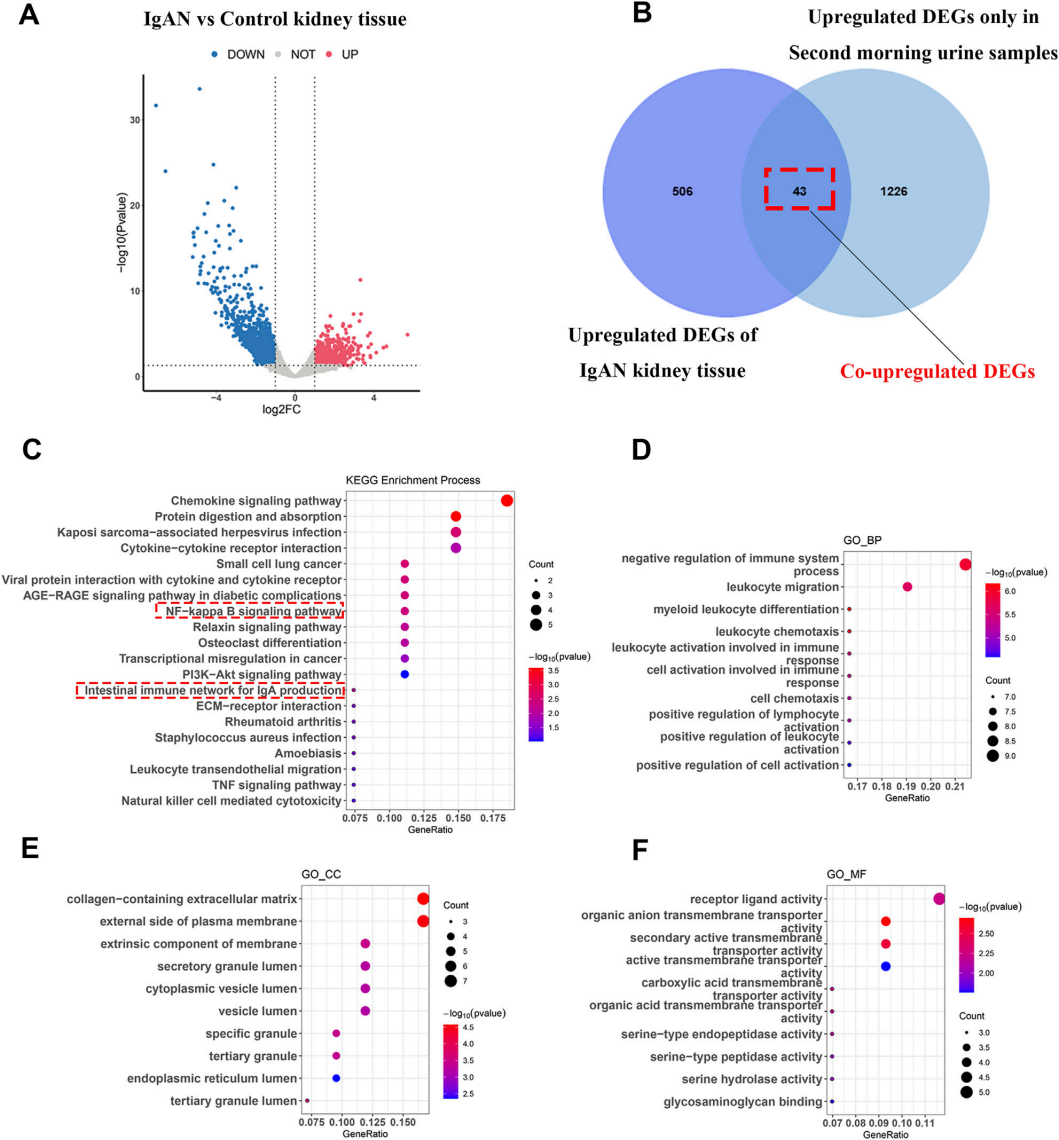
Functional insights and renal origins of commonly upregulated genes in second morning urine samples from IgAN patients **(A)** Differential gene analysis in IgAN kidney tissue. **(B)** Overlap between differential genes identified in second morning urine and those in kidney tissue. **(C–F)** KEGG and GO enrichment analyses highlighting associations with immune and signaling pathways.

To further explore the role of second morning urine in reflecting renal status, we analyzed IgAN-related datasets (GSE210098 and GSE141295) and identified 549 upregulated genes in IgAN renal tissues (Figure 3A). The complete list of differentially expressed genes (DEGs) can be found in Supplementary File 3. Intersection analysis with the upregulated genes in second morning urine revealed 43 genes that were upregulated in both the second morning urine and renal tissue of IgAN patients (Figure 3B), including genes such as *CD86*, *MMP9*, and *COL4A1* (Supplementary Table 3). These genes may directly mirror the pathological state of the kidney in IgAN.

KEGG enrichment analyses of these 43 commonly upregulated genes indicated that IgAN is not only associated with the intestinal immune network involving IgA production but also with chemokine signaling pathways, cytokine-cytokine receptor interactions, and the Nucleal factor kappaB (NF-κB) signaling pathway (Figure 3C). GO analysis further revealed that these genes are involved in critical biological processes such as negative regulation of the immune system, leukocyte migration, and myeloid leukocyte differentiation (Figure 3D), as well as being closely associated with cellular structures like the collagen-containing extracellular matrix, the external side of the plasma membrane, and membrane external components (Figure 3E). Functionally, ligand-receptor activity and organic anion transmembrane transporter activity were significantly enriched, highlighting their relevance to IgAN (Figure 3F).

### Machine learning identifies *TYROBP* and *HCK* as core genes for IgAN diagnosis

**FIGURE 4:**
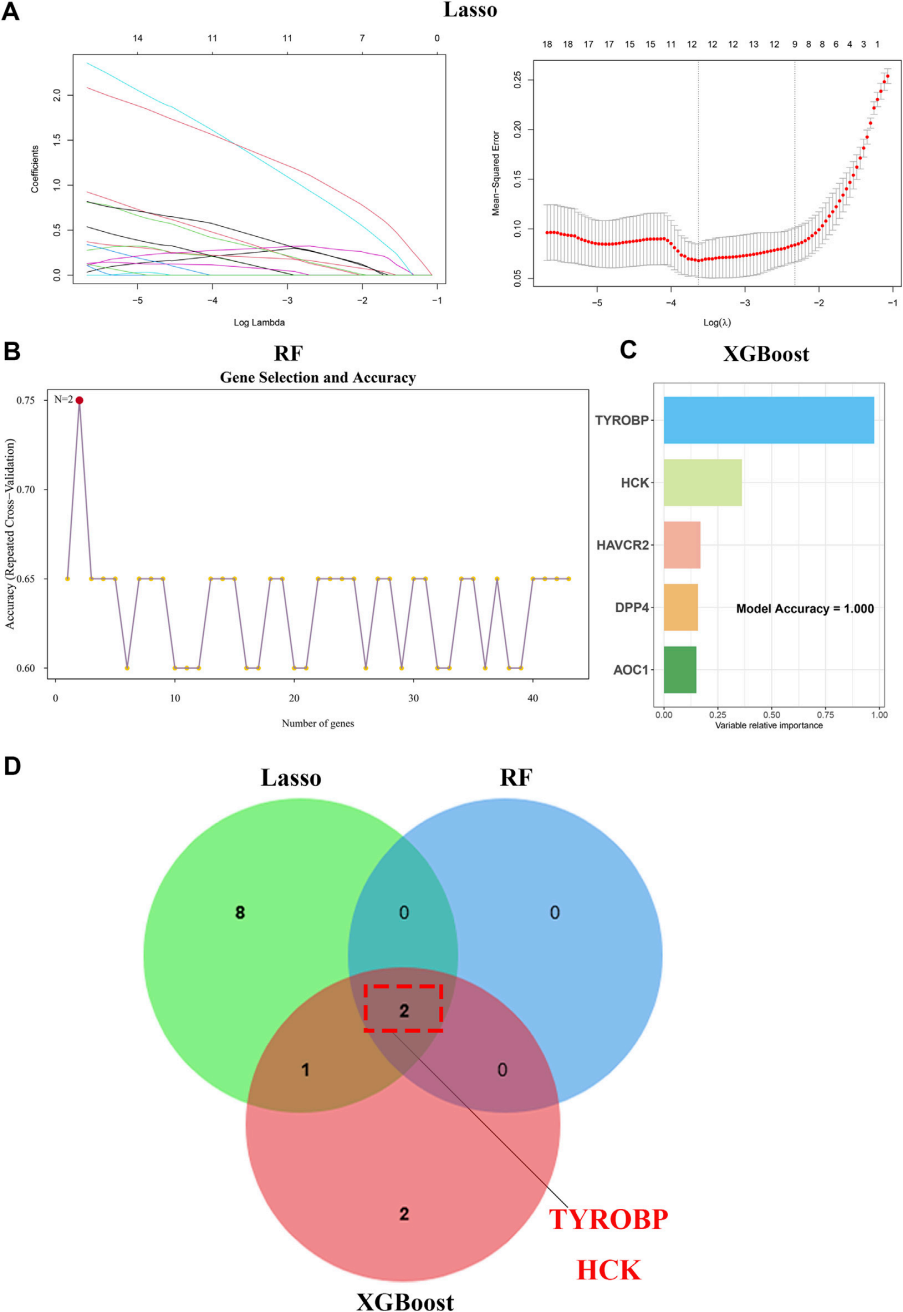
Machine learning unveils TYROBP and HCK as core IgAN biomarkers **(A)** Lasso regression identifies key genes from 43 upregulated genes in second-morning urine samples. **(B)** Random forest analysis further refines gene selection, pinpointing pivotal biomarkers. **(C)** XGBoost algorithm enhances feature importance, validating key genes for IgAN. **(D)** Venn diagram illustrates *TYROBP* and *HCK* as core diagnostic and prognostic markers for IgAN.

We utilized a comprehensive dataset encompassing 43 genes consistently upregulated in second morning urine samples and renal tissue samples to conduct machine learning-based feature selection and develop a robust diagnostic model. We began our analysis with Lasso regression (Figure 4A), a technique that facilitated regularization and helped isolate a subset of the most significant genes. This was followed by the application of random forest (Figure 4B) and XGBoost algorithms (Figure 4C), both of which are renowned for their ability to manage high-dimensional data and reveal intricate feature interactions.

Through these advanced analytical methods, we honed in on two pivotal genes: *TYROBP* and *HCK* (Figure 4D). These genes were subsequently employed to construct a diagnostic model, aimed at improving diagnostic precision and reliability.

### Single-cell resolution analysis highlights *TYROBP* and *HCK* expression in monocyte-macrophages in IgAN

**FIGURE 5:**
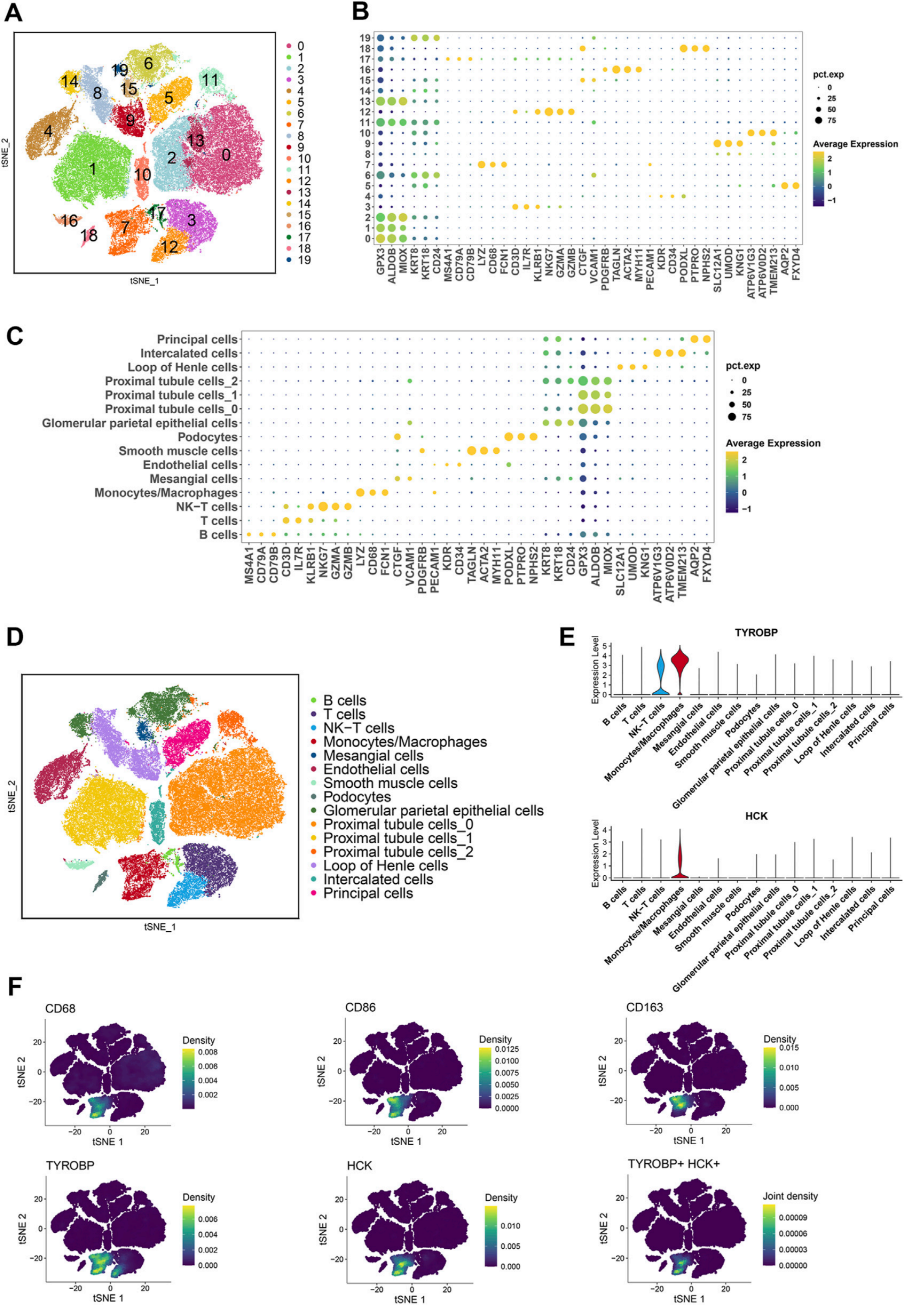
Single-cell transcriptomic profiling reveals distinct cellular clusters and marker gene expression patterns **(A, D)** Clustering of 70,299 cells from publicly available scRNA-seq datasets (GSE131685, GSE171314, GSE140989, GSE127136) into 20 distinct clusters, categorized into 15 different cell types. **(B, C)** Bubble plots illustrating the expression of marker genes across the 20 clusters and 15 cell types. **(E)** Violin plots showing high expression of *TYROBP* in monocyte-macrophages and NK-T cells, and high expression of *HCK* specifically in monocyte-macrophages. **(F)** Density plots further confirming the elevated expression of *TYROBP* and *HCK* in monocyte-macrophages.

To investigate the expression of *TYROBP* and *HCK* at single-cell resolution, we analyzed multiple scRNA-seq datasets from the Gene Expression Omnibus (GSE131685, GSE171314, GSE140989, GSE127136). After batch effect correction and principal component analysis (PCA), 70,299 cells were clustered into 20 distinct clusters and categorized into 15 different cell types (Figures 5A, D). Marker genes were sourced from the literature (Liao et al., 2020; Tang et al., 2021; Chen et al., 2024; Menon et al., 2020) and the CellMarker 2.0 database (Hu et al., 2022). Bubble plots illustrate the expression of these marker genes across 20 clusters and 15 cell types (Figures 5B, C).

Violin plots reveal that *TYROBP* is highly expressed in both monocyte-macrophage and NK-T cell populations, whereas *HCK* is predominantly expressed in the monocyte-macrophage cluster (Figure 5E). Density plots further corroborate the high expression of *TYROBP* and *HCK* specifically in monocyte-macrophages (Figure 5F).

### Distinct expression patterns of *TYROBP* and *HCK* in monocyte-macrophage subpopulations suggest their roles in IgAN progression

**FIGURE 6:**
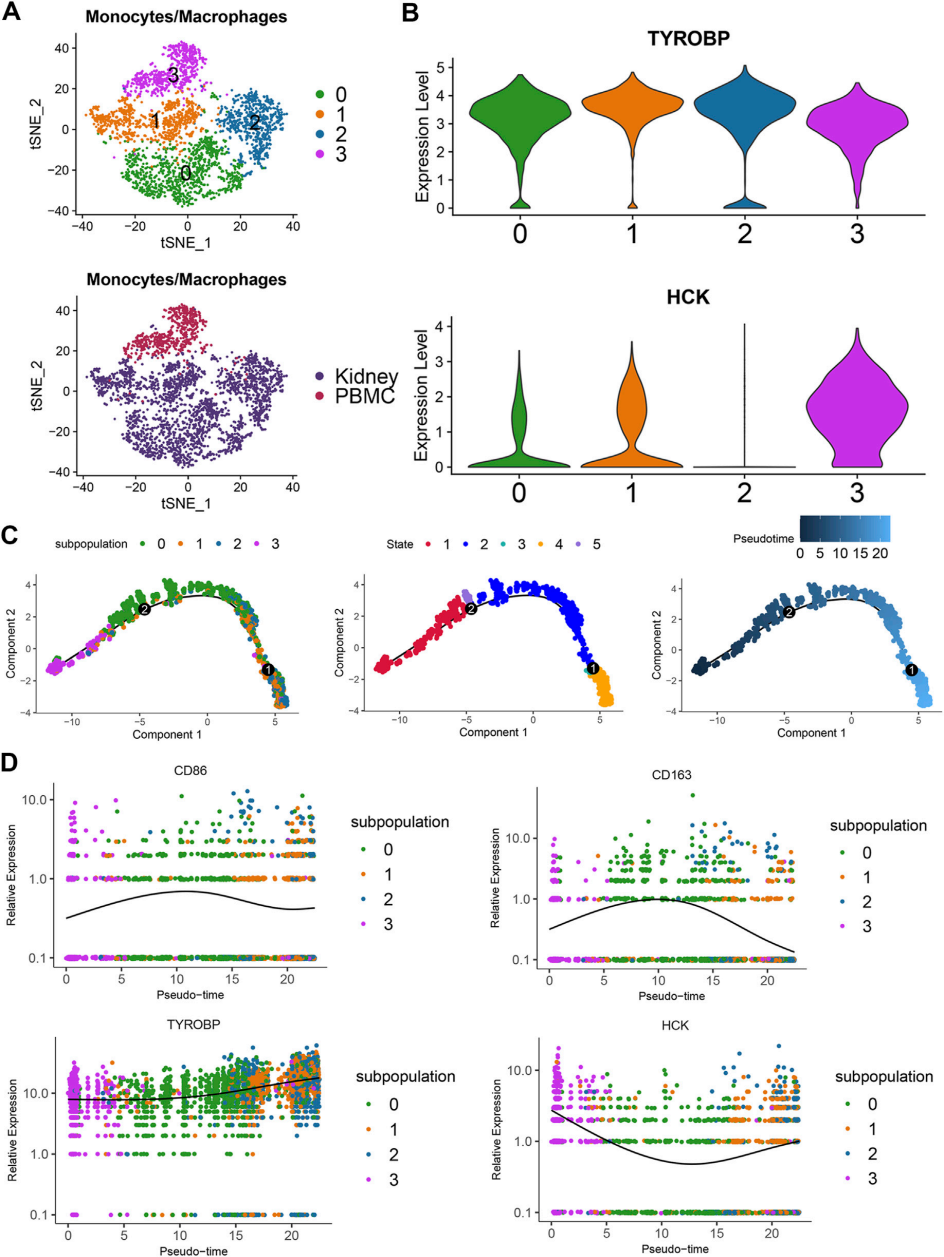
Differential expression and developmental dynamics of *TYROBP* and *HCK* in monocyte-macrophage subpopulations **(A)** t-SNE clustering of 3,676 monocyte-macrophage cells into four subgroups, with three of these subgroups primarily consisting of peripheral blood monocytes. **(B)** Violin plots showing that *TYROBP* is highly expressed in all subgroups, whereas *HCK* is predominantly expressed in subgroups 0, 1, and 3. **(C)** Pseudotime analysis indicates that the three subgroups predominantly composed of peripheral blood monocytes represent the developmental starting point, with cells transitioning from state 1 to state 4 along the pseudotime axis. **(D)** *TYROBP* maintains high and progressively increasing expression along the developmental trajectory, while *HCK*’s expression pattern inversely correlates with macrophage activation, suggesting a potential association with the activity state of monocyte-macrophages.

To further investigate the expression patterns of *TYROBP* and *HCK* in monocyte-macrophages, we analyzed 3,676 monocyte-macrophage cells and performed subpopulation analysis. t-SNE clustering divided the cells into four subpopulations, with subpopulation 3 predominantly comprising peripheral blood monocytes (Figure 6A). Violin plots reveal that *TYROBP* is highly expressed across all subpopulations, while *HCK* is predominantly expressed in subpopulations 0, 1, and 3 (Figure 6B).

Pseudotime analysis indicated that subpopulation 3 primarily consisting of peripheral blood monocytes represent the developmental starting point. Cells were classified into five states along a pseudotime trajectory, transitioning from state 1 through state 5, and gradually shifting towards state 4, reflecting dynamic changes in monocyte-macrophage states (Figure 6C). *TYROBP* maintains high expression throughout the developmental trajectory and shows an increasing trend over time (Figure 6D). This suggests that *TYROBP* may be a critical driver in the progression of IgAN. Interestingly, *HCK* exhibits an expression pattern inversely related to macrophage activation, indicating a possible association with the activity state of monocyte-macrophages (Figure 6D).

These findings underscore the involvement of TYROBP and HCK in the monocyte-macrophage lineage and their potential roles in IgAN pathogenesis and progression. Scatter plots further revealed positive correlations between TYROBP and monocyte-macrophage marker genes, including CD68, CD86, and CD163, within the monocyte-macrophage subpopulations. A similar correlation pattern was observed for HCK. Both TYROBP and HCK were also positively correlated with NFKBIA, indicating a potential link between monocyte-macrophages and the NF-κB signaling pathway in the context of IgAN (Supplementary Figure 1).

### Pronounced upregulation of TYROBP and HCK in IgAN renal tissues underscores their potential as diagnostic biomarkers

**FIGURE 7:**
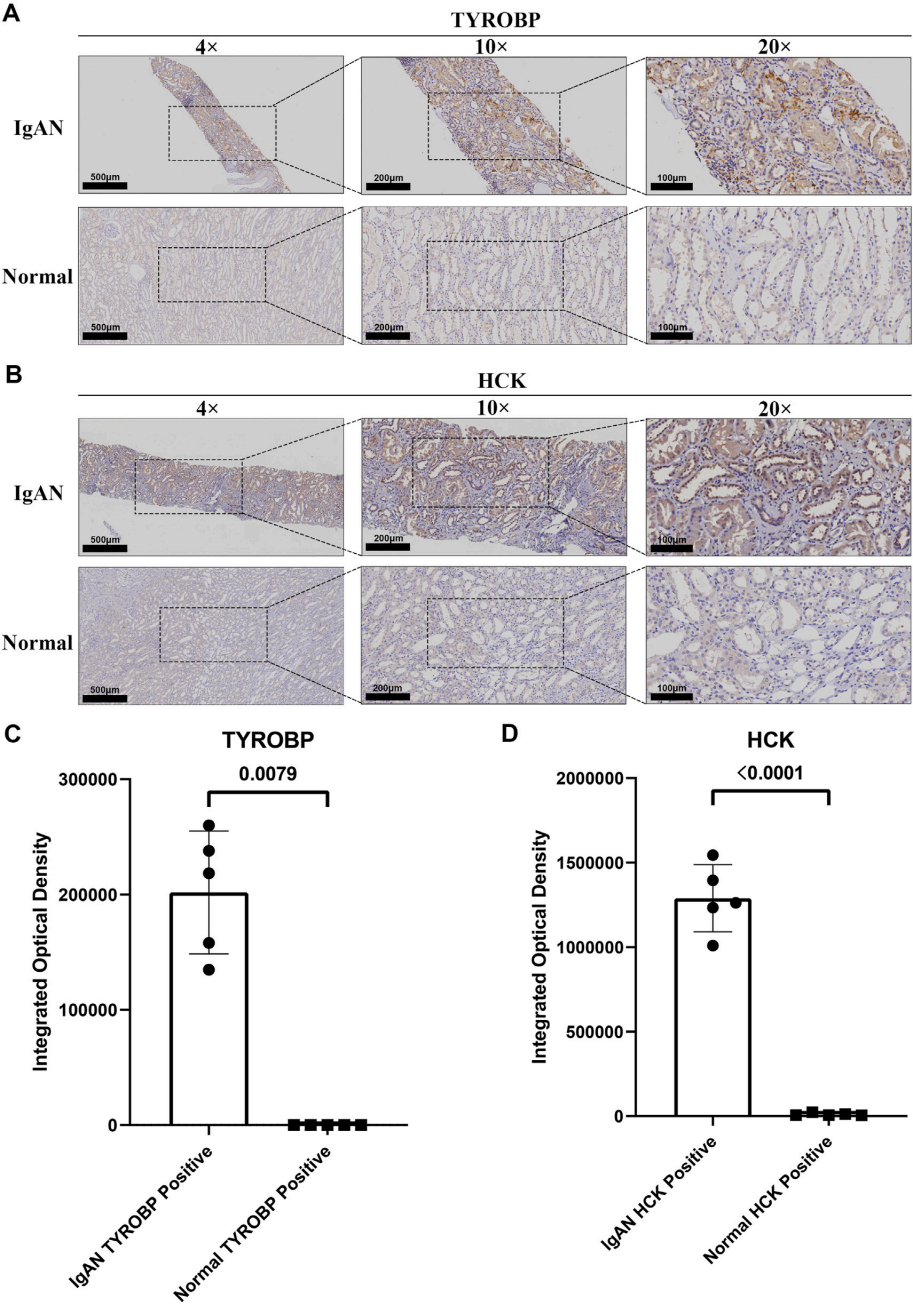
Elevated expression of *TYROBP* and *HCK* in IgAN renal tissues compared to normal kidney tissues. **(A)** Representative IHC staining of TYROBP in IgAN renal tissues and normal controls, demonstrating its upregulation in IgAN. Images are shown at 4×, ×10, and ×20 magnification. **(B)** Representative IHC staining of HCK in IgAN renal tissues and normal controls, revealing its increased expression in IgAN. Images are presented at 4×, ×10, and ×20 magnification. **(C)** Quantitative analysis of integrated optical density (IOD) for TYROBP in IgAN and normal kidney tissues. The IOD of TYROBP-positive areas in IgAN tissues is significantly higher than in normal tissues, confirming its upregulation. **(D)** Quantitative analysis of integrated optical density for HCK in IgAN and normal kidney tissues. The IOD of HCK-positive areas in IgAN tissues is significantly elevated compared to normal tissues, supporting its enhanced expression in IgAN.

IHC staining confirmed the marked upregulation of TYROBP in renal tissues from IgAN patients compared to normal controls (Figure 7A). TYROBP exhibited significantly increased staining intensity in IgAN samples, characterized by a higher density and number of positively stained cells. Similarly, HCK staining was markedly enhanced in IgAN renal tissues relative to normal samples (Figure 7B). These findings highlight the pivotal roles of TYROBP and HCK in IgAN pathophysiology and their potential as diagnostic biomarkers. Quantitative analysis of five randomly selected fields from the IHC images further validated these observations. Integrated optical density (IOD) analysis revealed that the IOD of TYROBP-positive areas in IgAN tissues was significantly greater than in normal tissues, with a statistically significant difference (Figure 7C). A comparable pattern was observed for HCK (Figure 7D).

To ensure staining specificity, IHC negative control experiments were conducted using secondary antibodies without primary antibodies, applied to both IgAN and normal renal tissues. As shown in Supplementary Figure 2, no discernible staining was observed under these conditions, confirming the specificity of the TYROBP and HCK staining patterns observed in Figures 7A, B. Additional validation from the Human Protein Atlas database revealed low baseline expression of TYROBP and HCK in normal kidney tissues (Supplementary Figure 3). This stark contrast emphasizes that their elevated expression is a distinct characteristic of IgAN, rather than a general feature of renal pathology.

Collectively, these findings highlight the potential of TYROBP and HCK as diagnostic markers for IgAN. Their distinct upregulation in IgAN renal tissues, compared to normal tissues, underscores their relevance to the disease’s underlying pathophysiology. Further research is warranted to elucidate their mechanistic roles in IgAN progression, which may provide novel insights into disease biology and inform the development of targeted therapeutic strategies.

### ROC analysis demonstrates high diagnostic accuracy of *TYROBP* and *HCK* for IgAN in test and validation sets

**FIGURE 8:**
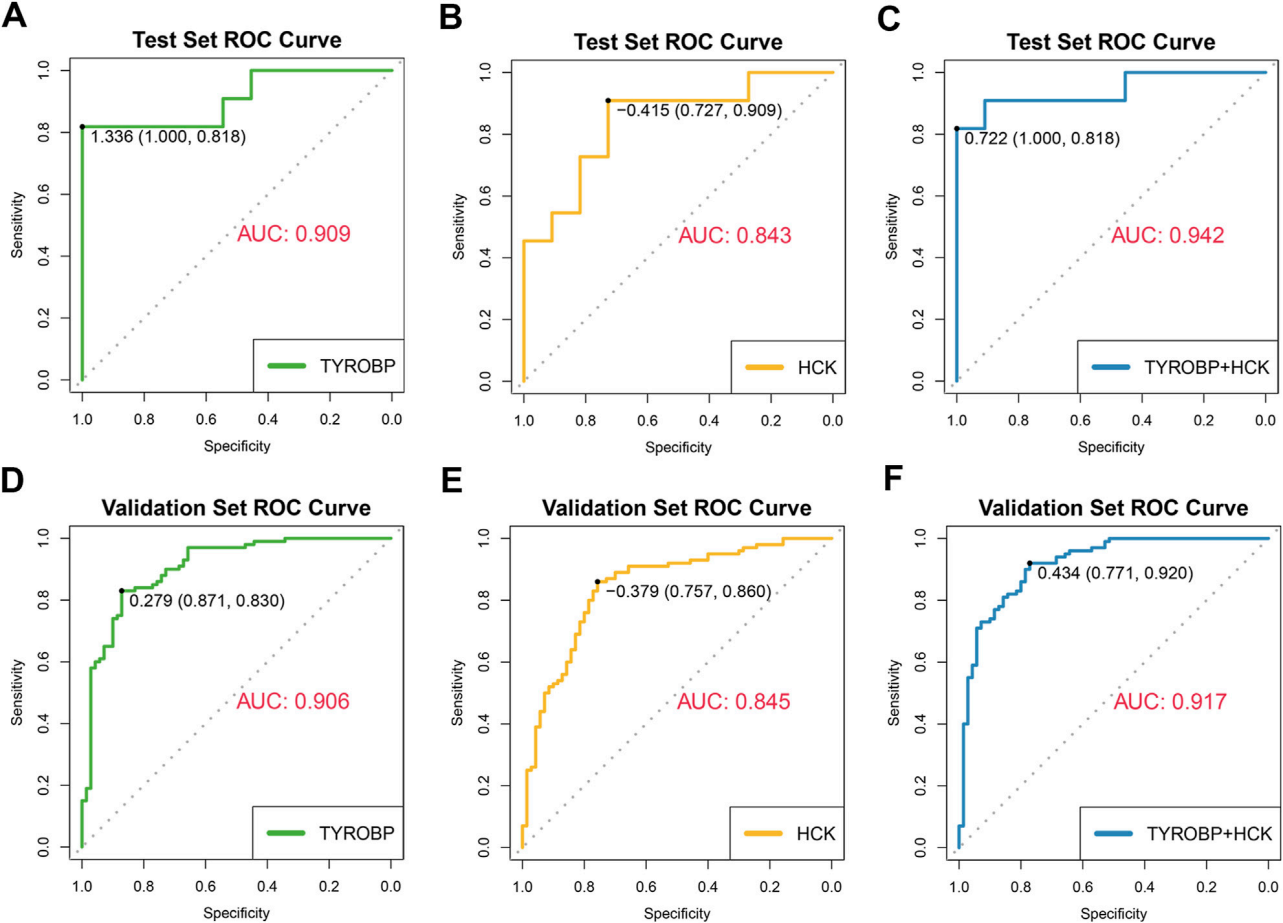
Diagnostic efficacy of *TYROBP* and *HCK* in IgAN: ROC curves and AUC values in test and validation sets. **(A, B)** ROC curves for *TYROBP* and *HCK* in the test set, showing AUC values of 0.909 and 0.843, respectively, indicating strong diagnostic performance. **(C)** Combined ROC curve for *TYROBP* and *HCK* in the test set, achieving an AUC of 0.942, demonstrating enhanced diagnostic accuracy. **(D, E)** ROC curves for *TYROBP* and *HCK* in the validation sets, with AUCs of 0.906 and 0.845, respectively, confirming robustness across external datasets. **(F)** Combined ROC curve for *TYROBP* and *HCK* in the validation sets, with an AUC of 0.917, highlighting the biomarkers’ potential for reliable early diagnosis and clinical management of IgAN.

We assessed the diagnostic performance of the core genes *TYROBP* and *HCK* using receiver operating characteristic (ROC) curves, with second-morning urine data serving as the test set (11 IgAN patients and 11 healthy controls) and GEO datasets (GSE37463, GSE93798, GSE99340, GSE104948) as external validation sets (100 IgAN patients and 70 healthy controls). *TYROBP* exhibited strong diagnostic capability, achieving an area under the curve (AUC) of 0.909 in the test set and 0.906 in the validation set (Figures 8A, D). Similarly, *HCK* demonstrated solid performance, with an AUC of 0.843 in the test set and 0.845 in the validation set (Figures 8B, E). When combined, *TYROBP* and *HCK* improved diagnostic accuracy, reaching an AUC of 0.942 in the test set and 0.917 in the validation set (Figures 8C, F).

These results highlight the robust potential of *TYROBP* and *HCK* as non-invasive biomarkers for diagnosing IgAN using second-morning urine samples. The strong performance across both test and validation sets suggests that these biomarkers could offer a reliable and effective tool for early diagnosis and clinical management of IgAN.

### *TYROBP* and *HCK* expression correlates with renal function decline and disease progression in IgAN patients

**FIGURE 9:**
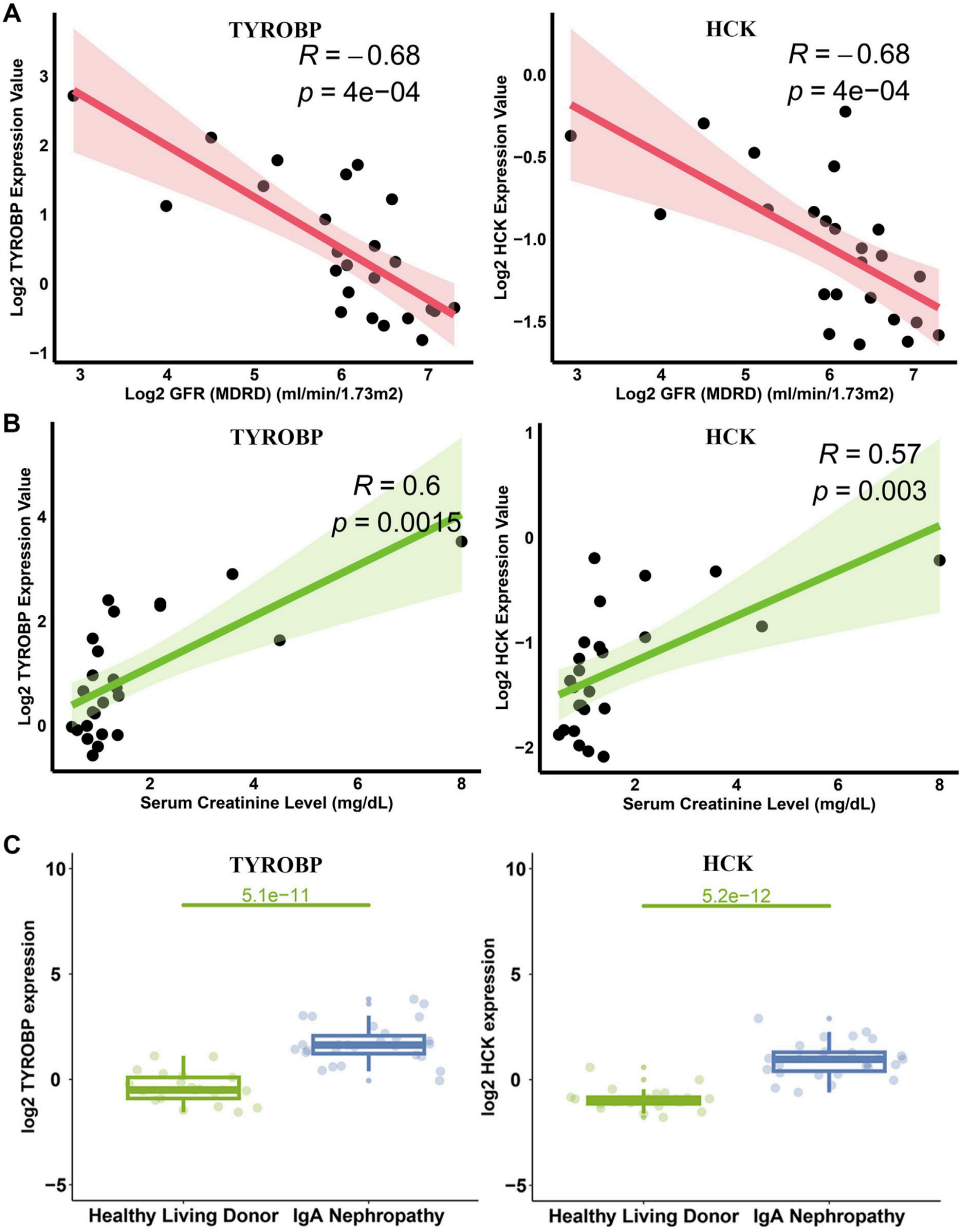
Correlation of *TYROBP* and *HCK* with GFR and Scr in IgAN. **(A)** Scatter plots depicting the inverse correlations of *TYROBP* and *HCK* with GFR in IgAN patients. **(B)** Scatter plots showing positive correlations of *TYROBP* and *HCK* with Scr levels. **(C)** Expression analysis from the Nephroseq database revealing significantly elevated levels of *TYROBP* and *HCK* in IgAN patients compared to controls.

To elucidate the clinical significance of *TYROBP* and *HCK* in IgAN, we analyzed their correlation with key clinical parameters using data from the Nephroseq v5 database. Scatter plot analyses revealed a strong inverse correlation between *TYROBP* expression and glomerular filtration rate (GFR) in IgAN patients (R = −0.68, *p* = 4e-04) (Figure 9A). Similarly, *HCK* expression showed a significant negative correlation with GFR (R = −0.68, *p* = 4e-04) (Figure 9A). In parallel, *TYROBP* levels were positively correlated with serum creatinine (R = 0.6, *p* = 0.0015) (Figure 9B), and *HCK* demonstrated a similar positive association with serum creatinine (R = 0.57, *p* = 0.003) (Figure 9B).

These findings underscore the potential of *TYROBP* and *HCK* as biomarkers for monitoring IgAN progression, given their associations with both decreased renal function and increased serum creatinine levels. Further expression analysis confirmed that both *TYROBP* and *HCK* levels were significantly elevated in IgAN patients compared to healthy controls (Figure 9C), reinforcing their potential as key indicators in the pathophysiology and clinical progression of the disease.

### Strong drug-protein interactions of TYROBP and HCK with IgAN therapies

In this study, we also employed molecular docking techniques to examine the interactions between key clinical drugs used to slow the progression of IgAN and the proteins TYROBP and HCK. The drugs analyzed included RAS inhibitors (RASi) (Yang et al., 2018; Sugiura et al., 2021), selective sodium-glucose co-transporter 2 inhibitors (SLTG2i) (Dong et al., 2023), Sparsentan (Barratt et al., 2023a; Selvaskandan et al., 2024), and Budesonide (Barratt et al., 2023b; Selvaskandan et al., 2024; Wang J. et al., 2024). Sacubitril valsartan sodium hydrate is a RAS inhibitor that helps lower blood pressure and improve heart function. Dapagliflozin, a selective SGLT2 inhibitor, works by preventing glucose reabsorption in the kidneys, offering renal protection. Sparsentan is a dual-action drug that blocks both endothelin and angiotensin II receptors, targeting renal health. Budesonide is a corticosteroid that reduces inflammation in kidney diseases like IgAN.

**FIGURE 10:**
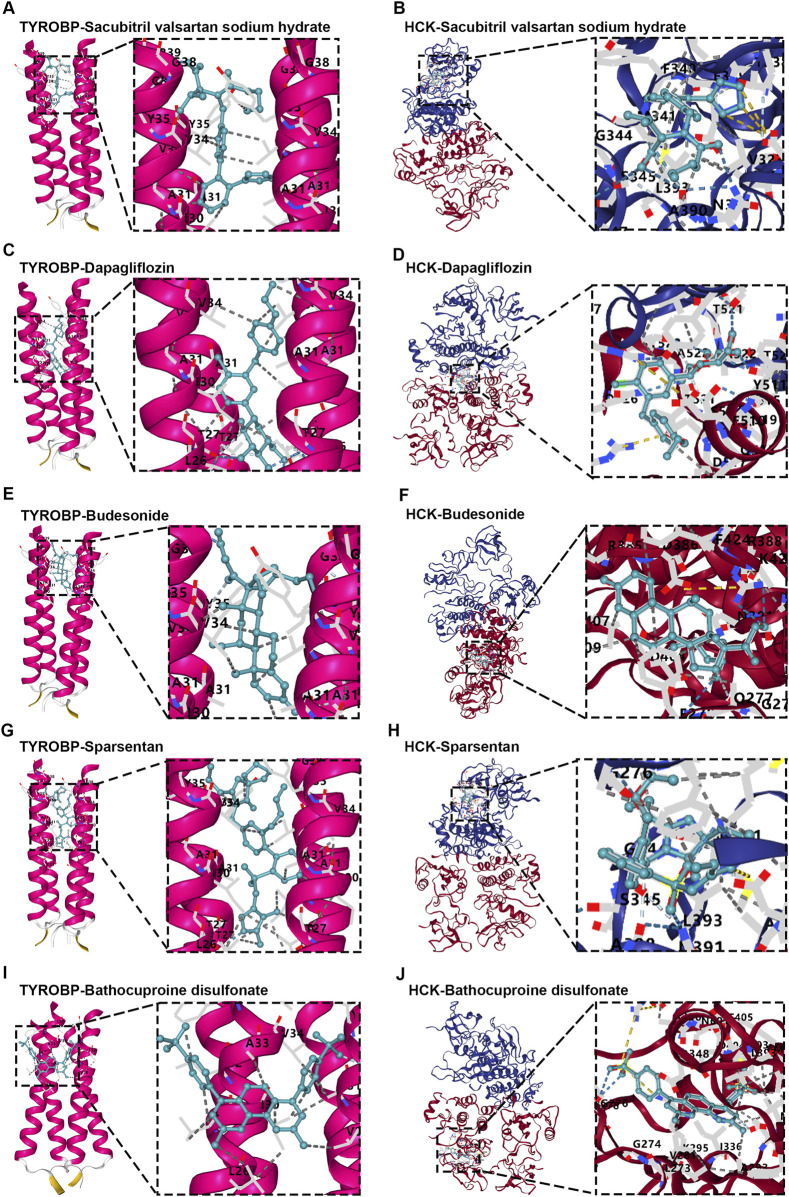
Molecular docking analysis of diagnostic proteins TYROBP and HCK with small molecules. **(A, B)** Sacubitril valsartan sodium hydrate docked with TYROBP and HCK, showing strong binding affinities indicative of potential modulation of disease pathways. **(C, D)** Dapagliflozin interactions with TYROBP and HCK, exhibiting the lowest binding free energy among tested compounds, highlighting renal protective potential. **(E, F)** Budesonide docked with TYROBP and HCK, demonstrating interactions relevant to inflammation control. **(G, H)** Sparsentan binding to TYROBP and HCK underscores its dual receptor blockade properties, with implications for renal health management. **(I, J)** Bathocuproine disulfonate docked with TYROBP and HCK, emerging as a novel therapeutic candidate with promising binding energies, particularly notable for HCK.

Molecular docking analyses revealed that all tested drugs exhibited strong binding affinities for both TYROBP and HCK, with the lowest recorded binding free energies for each compound. Sacubitril valsartan sodium hydrate, for instance, showed binding free energies of −9.1 kcal/mol for TYROBP (Figure 10A) and −8.7 kcal/mol for HCK (Figure 10B). Similarly, Dapagliflozin displayed binding free energies of −10.4 kcal/mol for TYROBP (Figure 10C) and −8.2 kcal/mol for HCK (Figure 10D), while Sparsentan showed binding free energies of −9.5 kcal/mol for TYROBP (Figure 10E) and −8.6 kcal/mol for HCK (Figure 10F). Budesonide demonstrated binding free energies of −7.9 kcal/mol for TYROBP (Figure 10G) and −8.3 kcal/mol for HCK (Figure 10H). In addition, utilizing the DSigDB, we identified Bathocuproine disulfonate as a novel compound that exhibited promising binding to both TYROBP and HCK, with binding free energies of −7.1 kcal/mol for TYROBP (Figure 10I) and −10.7 kcal/mol for HCK (Figure 10J).

These results indicate that all clinically relevant drugs analyzed—Sacubitril valsartan sodium hydrate, Dapagliflozin, Sparsentan, and Budesonide—demonstrate strong binding affinity to the diagnostic proteins TYROBP and HCK, as evidenced by the lowest free energies. Additionally, Bathocuproine disulfonate emerges as a potential novel modulator of IgAN progression, highlighting its promise as a therapeutic agent. The robust interactions between these drugs and key diagnostic proteins underscore the critical role of TYROBP and HCK in IgAN pathophysiology, suggesting that targeting these proteins could enhance therapeutic efficacy. The identification of Bathocuproine disulfonate as a potential new therapeutic candidate presents exciting opportunities for the development of more effective treatment strategies, with the potential to improve patient outcomes in IgAN management.

## Discussion

This study identifies *TYROBP* and *HCK* as pivotal non-invasive urinary biomarkers for IgAN, demonstrating their potential for improving IgAN diagnosis. Our comprehensive analysis of urine and kidney tissue samples, combined with bulk and scRNA-seq, revealed distinct expression patterns of these biomarkers in monocyte-macrophages, with *TYROBP* progressively increasing along the pseudotime trajectory, suggesting its role in disease progression. ScRNA-seq also showed that *HCK* inversely correlates with macrophage polarization, hinting at its involvement in modulating immune responses. These findings underscore the relevance of urinary transcriptomics in IgAN diagnosis, with our diagnostic models showing robust performance validated across multiple cohorts. These results pave the way for new, non-invasive diagnostic and therapeutic approaches.

*TYROBP* is a transmembrane signaling polypeptide known to regulate immune cell functions through interactions with various surface receptors on immune cells (Hamerman et al., 2009; Haure-Mirande et al., 2022). It is known to mediate inflammatory responses by modulating cytokine production (Turnbull et al., 2005) and to induce the proliferation and survival of macrophages (Otero et al., 2009). In other disease contexts, such as neuroinflammation (Zhou et al., 2023) and osteoporosis (Paloneva et al., 2003), deficiencies in *TYROBP* signaling lead to microglial dysfunction and impaired osteoclast differentiation. The interaction of *TYROBP* with its receptor, TREM-1, amplifies inflammatory responses through synergy with TLR signaling, initiating intracellular signaling cascades (Carrasco et al., 2019). Moreover, the TYROBP-SYK pathway promotes macrophage secretion of TGF-β, exacerbating inflammation (Takamiya et al., 2013). These mechanisms suggest that *TYROBP* may contribute to the progression of IgAN by modulating immune responses and inflammation. Despite its well-documented role in other diseases, including renal cell carcinoma (Guo et al., 2023), osteosarcoma (Li et al., 2022; Wei et al., 2023), and breast cancer (Shabo et al., 2013), its precise role in IgAN remains unclear, warranting further investigation.

*HCK*, a member of the SRC family kinases (SFKs), is a non-receptor protein tyrosine kinase primarily expressed in myeloid and B lymphoid cell lineages (Poh et al., 2015; Parsons and Parsons, 2004). It plays a crucial role in regulating various cell signaling pathways, particularly in macrophage polarization, migration, and proliferation (Poh et al., 2015; Xiao et al., 2008; Cougoule et al., 2010). *HCK* has been implicated in the pathogenesis of renal fibrosis (Wang et al., 2018), a common outcome in IgAN, and is known to modulate macrophage functions such as inflammatory polarization and migration. Previous studies have highlighted its involvement in fibrosis-related chronic diseases, including pulmonary fibrosis (Ernst et al., 2002; Kanderova et al., 2022) and renal fibrosis (Wei et al., 2017). Recent findings suggest that *HCK* contributes to renal fibrosis by regulating macrophage activity (Chen M. et al., 2023). ScRNA-seq analysis from IgAN kidney tissues reveals that *HCK* is predominantly expressed in mononuclear macrophages, pointing to its potential role in IgAN pathology. Although its specific functional roles in IgAN remain to be further explored, these findings suggest that *HCK* may be a key modulator of immune responses and fibrosis in IgAN.

It is noteworthy that molecular docking analyses indicate that frontline therapies for IgAN, including Sacubitril valsartan sodium hydrate (RAS inhibitor), Dapagliflozin (SGLT2 inhibitor), Sparsentan, and Budesonide, exhibit strong binding affinities to TYROBP and HCK. RAS inhibitors reduce blood pressure and renal load by suppressing the renin-angiotensin system, thereby lowering proteinuria, enhancing renal function, and potentially slowing IgAN progression (Kunz et al., 2008). SGLT2 inhibitors protect kidney function by reducing intrarenal pressure and renal workload, thus alleviating proteinuria and delaying renal decline (Wheeler et al., 2021). Sparsentan, a dual endothelin and angiotensin II receptor antagonist, effectively lowers proteinuria and supports renal function, achieving outcomes similar to the RAS inhibitors (Kowala et al., 2004; Trachtman et al., 2020).

Budesonide, a kidney-targeted corticosteroid, mitigates renal injury progression by reducing proteinuria, and improving renal function (Smerud et al., 2011). As an immunosuppressant, Budesonide’s strong binding affinity to TYROBP and HCK aligns with its role in attenuating inflammation. Surprisingly, other agents—primarily acting to modulate renal filtration protein levels rather than inflammation—also exhibit robust binding interactions with TYROBP and HCK, proteins highly expressed in monocyte-macrophage cells. This finding suggests potential novel pathways warranting further mechanistic investigation to elucidate the nature of these drug-protein interactions.

Additionally, our study identified Bathocuproine disulfonate, a novel compound with strong binding affinities to both TYROBP and HCK. Known for selectively chelating monovalent copper ions (Cu⁺), Bathocuproine disulfonate stabilizes reactive copper ions that otherwise catalyze oxidative stress reactions, triggering cell damage (Zhang et al., 2022). By chelating copper ions, it may reduce oxidative stress and alleviate inflammatory responses in renal tissues, potentially slowing IgAN progression. Interestingly, Bathocuproine disulfonate has already been explored in cancer therapy research (Wang Y. et al., 2024), where its antioxidant and anti-inflammatory properties are being studied for therapeutic applications. This multifunctional profile highlights Bathocuproine disulfonate as a promising candidate for further investigation as a therapeutic agent, targeting TYROBP and HCK interactions to potentially benefit IgAN treatment.

Despite the novel insights provided by our study regarding the diagnosis and progression of IgAN, there are limitations. The cross-sectional design of our study limits the evaluation of the prognostic value of urinary *TYROBP* and *HCK*. Future research should consider prospective cohort studies with extended follow-up periods to better assess the prognostic significance of these biomarkers in IgAN. Additionally, the mechanisms by which *TYROBP* and *HCK* influence monocyte-macrophages in IgAN progression warrant further investigation.

Based on our analysis, this study identifies the upregulation of *TYROBP* and *HCK* in second morning urine, revealing distinct molecular features of monocyte-macrophages in IgAN. These findings offer new insights into the pathophysiology of IgAN. Importantly, our study highlights the significance of examining transcriptional changes in the urinary transcriptome, suggesting novel approaches for developing more effective non-invasive diagnostic and therapeutic strategies.

## Conclusion

This study significantly advances our understanding of the urinary transcriptomic landscape in IgAN patients, offering novel diagnostic and therapeutic insights. Through bulk RNA-seq, we identified *TYROBP* and *HCK* as key non-invasive urinary biomarkers. scRNA-seq further revealed the renal cellular origins of these biomarkers, linking them to disease-specific pathways. IHC confirmed their protein-level expression in renal tissues, while molecular docking demonstrated strong binding affinities with existing IgAN treatments, highlighting their therapeutic potential. These findings deepen our understanding of IgAN’s molecular mechanisms and contribute to the development of precision diagnostic and therapeutic strategies for the disease.

## Data Availability

The original contributions presented in the study are included in the article/Supplementary Material, further inquiries can be directed to the corresponding authors.
