# Supplementary material for: Urinary TYROBP and HCK as genetic biomarkers for non-invasive diagnosis and therapeutic targeting in IgA nephropathy
Source: Front Genet. 2024 Dec 24;15:1516513. doi: 10.3389/fgene.2024.1516513 (PMC11703869; doi:10.3389/fgene.2024.1516513)
Supplement: Supplementary file 1 [file Table1.docx]

Supplementary Table 1

| **Dataset ID** | **Platform** | **Data Type** | **Tissue** | **IgAN Samples(n)** | **Control Samples(n)** |
| --- | --- | --- | --- | --- | --- |
| GSE141295 | GPL16791 | Bulk RNA | Kidney | 14 | 10 |
| GSE210098 | GPL24676 | Bulk RNA | Kidney | 17 | 0 |
| GSE37463 | GPL11670  GPL14663 | Microarray | Kidney | 27 | 27 |
| GSE93798 | GPL22945 | Microarray | Kidney | 20 | 22 |
| GSE99340 | GPL19109 | Microarray | Kidney | 26 | 0 |
| GSE104948 | GPL24120 GPL22945 | Microarray | Kidney | 27 | 21 |
| GSE131685 | GPL20795 | ScRNA | Kidney | 0 | 3 |
| GSE171314 | GPL20795 | ScRNA | Kidney | 4 | 1 |
| GSE140989 | GPL20301 | ScRNA | Kidney | 0 | 24 |
| GSE127136 | GPL20301 | ScRNA | Kidney | 13 | 6 |
|  |  |  | PBMC | 5 | 5 |

**Table S1:** This study incorporates datasets obtained from the Gene Expression Omnibus (GEO) database.
