# Supplementary figures and images for: Urinary TYROBP and HCK as genetic biomarkers for non-invasive diagnosis and therapeutic targeting in IgA nephropathy
Source: Front Genet. 2024 Dec 24;15:1516513. doi: 10.3389/fgene.2024.1516513 (PMC11703869; doi:10.3389/fgene.2024.1516513)

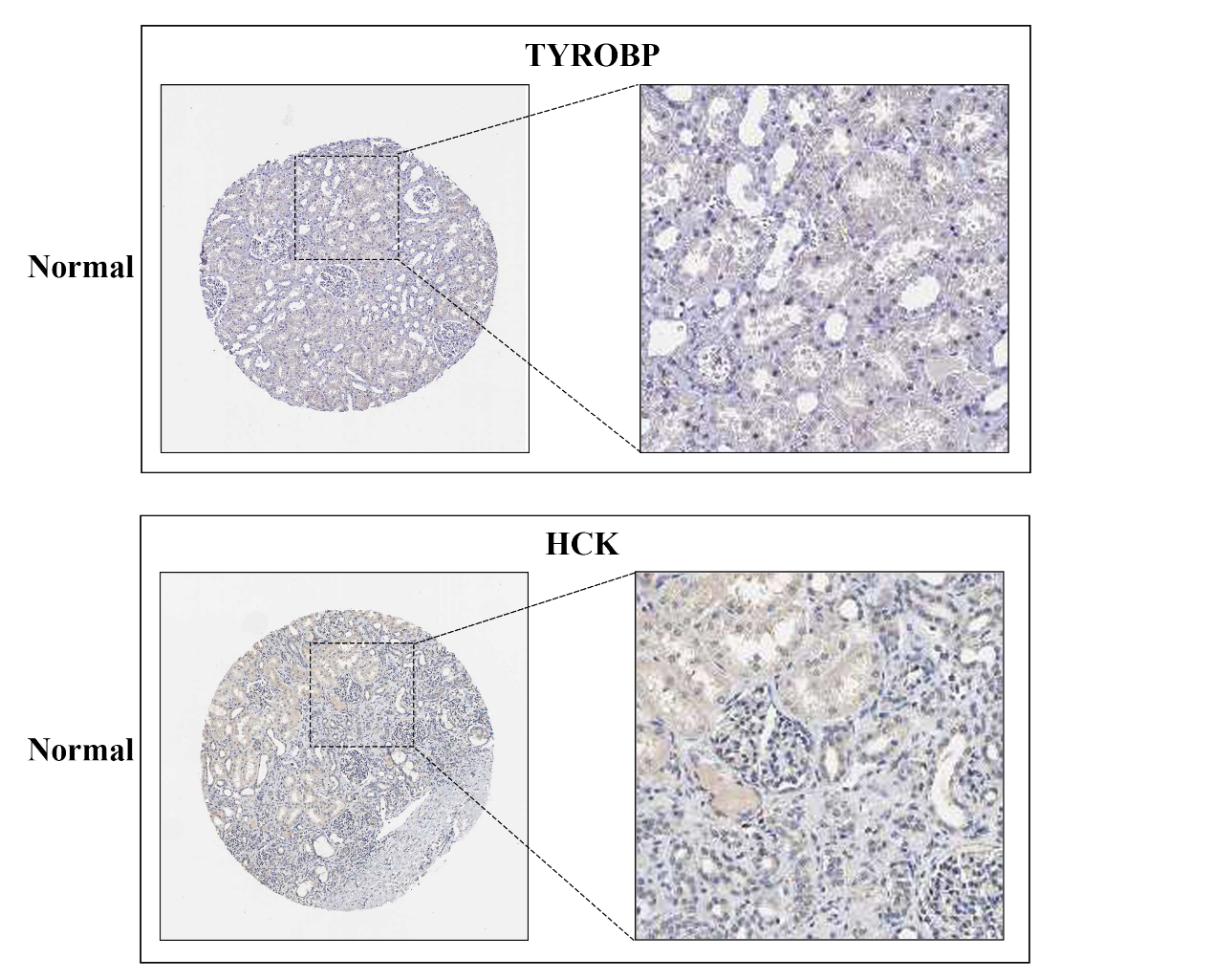


**Figure S3: IHC images of TYROBP andHCK in normal renal tissue from the HPA database.**

Supplement: Supplementary file 4 [file DataSheet3.docx]
