# Supplementary material for: Urinary TYROBP and HCK as genetic biomarkers for non-invasive diagnosis and therapeutic targeting in IgA nephropathy
Source: Front Genet. 2024 Dec 24;15:1516513. doi: 10.3389/fgene.2024.1516513 (PMC11703869; doi:10.3389/fgene.2024.1516513)
Supplement: Supplementary file 5 [file Table2.docx]

Supplementary Table1 2

| **Subject ID** | **Sex** | **Age** |
| --- | --- | --- |
| IgAN01 | Female | 65 |
| IgAN02 | Male | 31 |
| IgAN03 | Female | 36 |
| IgAN04 | Male | 47 |
| IgAN05 | Female | 27 |
| IgAN06 | Female | 30 |
| IgAN07 | Male | 37 |
| IgAN08 | Female | 24 |
| IgAN09 | Female | 32 |
| IgAN10 | Female | 47 |
| IgAN11 | Male | 41 |
| Control01 | Male | 40 |
| Control02 | Female | 36 |
| Control03 | Male | 33 |
| Control04 | Male | 36 |
| Control05 | Female | 31 |
| Control06 | Female | 73 |
| Control07 | Female | 24 |
| Control08 | Male | 54 |
| Control09 | Female | 45 |
| Control10 | Female | 46 |
| Control11 | Female | 31 |

**Table S2:** This study presents the baseline characteristics of IgAN patients and control groups included in the urinary bulk RNA sequencing analysis.
