# Supplementary material for: Urinary TYROBP and HCK as genetic biomarkers for non-invasive diagnosis and therapeutic targeting in IgA nephropathy
Source: Front Genet. 2024 Dec 24;15:1516513. doi: 10.3389/fgene.2024.1516513 (PMC11703869; doi:10.3389/fgene.2024.1516513)
Supplement: Supplementary file 6 [file Table3.docx]

Supplementary Table1 3

| **Project** | **Gene** |
| --- | --- |
| Co-upregulated genes | *CD86, CD84, CD163, MMP9, TMEM132A, SFRP2,*  *COL4A1, SLIT2, MS4A7, SYT11, LYPD1, COL4A2,*  *IGSF6, HCK, NFATC1, UCP2, TNFRSF9, GFRA2,*  *CDH2, GAS7, DPP4, SLC36A2, CXCL12, ADGRE2,*  *FAM124A, TYROBP, FCAR, TRAF1, GPR183, GGH,*  *SLC22A8, OSCAR, AOC1, HAVCR2, IFI30, SLC13A3,*  *CCR7, HK3, PLG, CCL19, ERFE, SORCS3, PIK3R5* |
| Lasso selected genes | *CCR7, CD84, CD86, ERFE, GFRA2, HAVCR2, HCK,*  *PLG, SFRP2, SLIT2, TYROBP* |
| RF selected genes  XGBoost selected genes | *TYROBP, HCK*  *TYROBP, HCK, HAVCR2, DPP4, AOC1* |

**Table S3:** Genes upregulated in second morning urine and renal tissue of IgAN, and those identified via integrative machine learning screening.
