# Supplementary material for: Urinary TYROBP and HCK as genetic biomarkers for non-invasive diagnosis and therapeutic targeting in IgA nephropathy
Source: Front Genet. 2024 Dec 24;15:1516513. doi: 10.3389/fgene.2024.1516513 (PMC11703869; doi:10.3389/fgene.2024.1516513)
Supplement: Supplementary file 7 [file DataSheet2.docx]

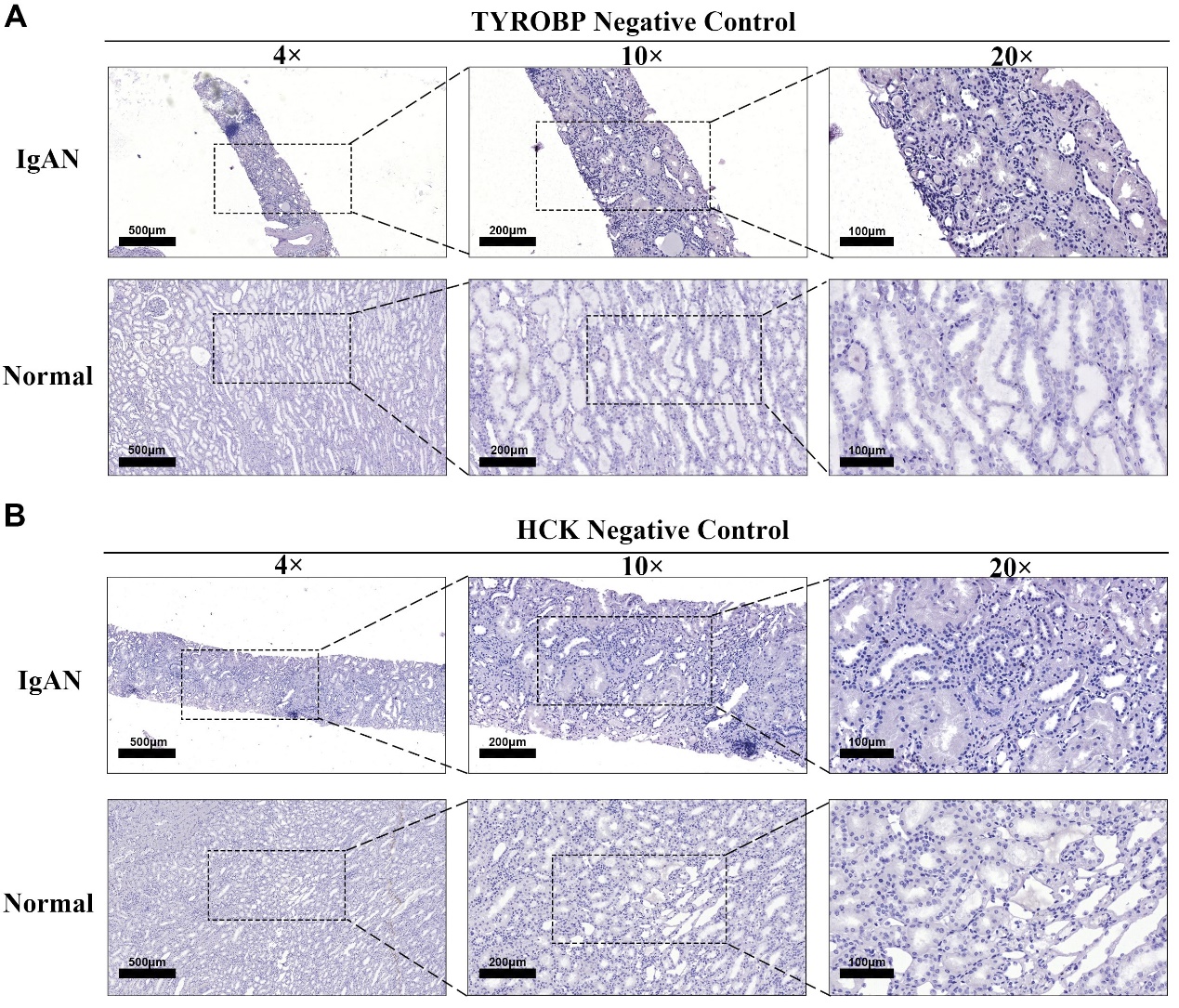


**Figure S2: IHC analysis of TYROBP and HCK expression in IgAN and normal kidney tissues, with negative controls included.**

(A) Negative controls (secondary antibody only, without primary antibody) in both IgAN and normal tissues confirm staining specificity. Images are displayed at magnifications of 4×, 10×, and 20×.

(B) Negative controls (secondary antibody only, without primary antibody) performed on both IgAN and normal tissues validate staining specificity. Images are shown at magnifications of 4×, 10×, and 20×.
