# Supplementary material for: Urinary TYROBP and HCK as genetic biomarkers for non-invasive diagnosis and therapeutic targeting in IgA nephropathy
Source: Front Genet. 2024 Dec 24;15:1516513. doi: 10.3389/fgene.2024.1516513 (PMC11703869; doi:10.3389/fgene.2024.1516513)
Supplement: Supplementary file 10 [file DataSheet1.docx]

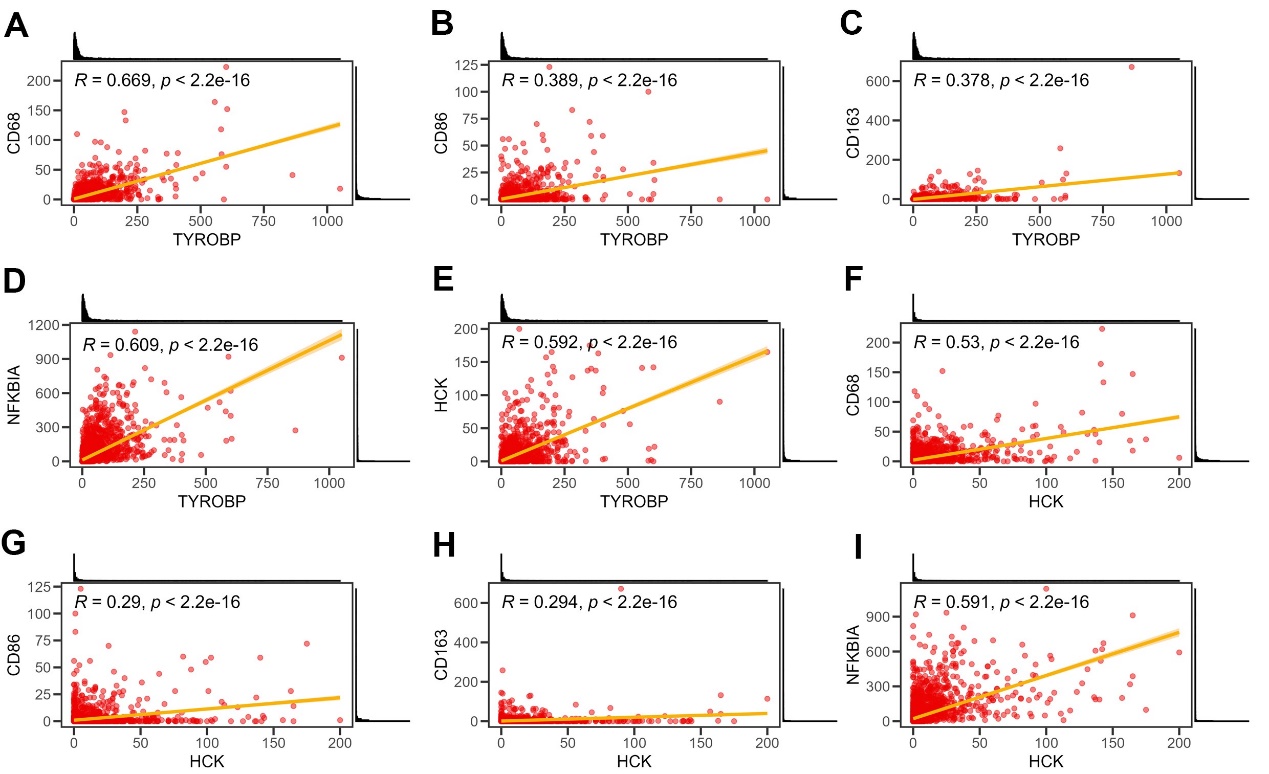


**Figure S1: Scatter plots illustrating expression correlations within monocyte-macrophage subpopulations.**

(A-I) Scatter plots show the expression correlations of *TYROBP* and *HCK* with *CD68*, *CD86*, *CD163*, and *NFKBIA* within monocyte-macrophage subpopulations.
